# Reirradiation − still navigating uncharted waters?^☆^

**DOI:** 10.1016/j.ctro.2024.100871

**Published:** 2024-10-02

**Authors:** Nicolaus Andratschke, Jonas Willmann, Ane L Appelt, Madalyne Day, Camilla Kronborg, Mariangela Massaccesi, Mahmut Ozsahin, David Pasquier, Primoz Petric, Oliver Riesterer, Dirk De Ruysscher, Joanne M Van der Velden, Matthias Guckenberger

**Affiliations:** a Department of Radiation Oncology, University Hospital of Zurich, University of Zurich, Switzerland; b Leeds Institute of Medical Research at St James’s, University of Leeds, UK; c Leeds Cancer Centre, Leeds Teaching Hospitals NHS Trust, UK; d Danish Centre for Particle Therapy, Aarhus University Hospital, Department of Clinical Medicine, Aarhus University, Aarhus, Denmark; e Department of Radiology, Radiation Oncology and Hematology, Fondazione Policlinico Universitario “A. Gemelli” IRCCS, Rome, Italy; f Hôpital Riviera-Chablais, Rennaz, Switzerland; g Academic Department of Radiation Oncology, Centre O Lambret, Lille, France; h University of Lille, Centrale Lille, CNRS, CRIStAL UMR 9189, Lille, France; i Cantonal Hospital Aarau, Aarau, Switzerland; j Maastricht University Medical Center+, Department of Radiation Oncology (Maastro), GROW School and Erasmus MC Cancer Institute, University Medical Center Rotterdam, the Netherlands; k Department of Radiation Oncology, University Medical Center Utrecht, Utrecht, the Netherlands

## Abstract

With the emergence of high-precision radiotherapy technologies such as stereotactic ablative radiotherapy (SABR), MR guided brachytherapy, image guided intensity modulated photon and proton radiotherapy and most recently daily adaptive radiotherapy, reirradiation is increasingly recognized as a viable treatment option for many patients. This includes those with recurrent, metastatic or new malignancies post initial radiotherapy. The primary challenge in reirradiation lies in balancing tumor control against the risk of severe toxicity from cumulative radiation doses to previously irradiated normal tissue.

Although technology for precise delivery has advanced at a fast pace, clinical practice of reirradiation still mostly relies on individual expertise, as prospective evidence is scarce, the level of reporting in clinical studies is not standardized and of low quality − especially with respect to cumulative doses received by organs at risk.

A recent ESTRO/EORTC initiative proposed a standardized definition of reirradiation and formulated general requirements for minimal reporting in clinical studies [1].

As a consequence we found it timely to convene for an international and interdisciplinary meeting with experts in the field to summarize the current evidence, identify knowledge gaps and explore which best practices can be derived for safe reirradiation. The meeting was held on 15.06.2023 in Zurich and was endorsed by the scientific societies SASRO, DEGRO and ESTRO. Here, we report on available evidence and research priorities in the field of reirradiation, as discussed during the meeting.

## Highlights

• Lack of high-quality evidence and standardized reporting for reirradiation studies.
• Scarcity of preclinical data on reirradiation efficacy and tissue toxicity recovery.
• Precision techniques including SABR or MR-guided brachytherapy advance reirradiation.
• Balancing tumor control and toxicity from cumulative radiation doses is challenging.
• Challenges include patient selection, dose assessment, and workflow optimization.

## Tumor and radiobiological considerations for reirradiation

Notably, there is a lack of preclinical data regarding the efficacy of radiotherapy on previously irradiated tumor cells. Consequently, the efficacy of reirradiation must be inferred from available clinical data. In the palliative setting, reirradiation has shown similar response rates in alleviating symptoms, as evidenced by a prospective randomized trial focusing on pain from spinal bone metastases [2] and a systematic review on thoracic reirradiation for hemoptysis, dyspnea, and bleeding [3]. In high-dose reirradiation scenarios, a positive dose–response relationship has been observed for lung SBRT [4], and several prospective randomized trials have demonstrated improvements in local control and progression-free survival [5], [6].

Regarding radiobiology of normal tissue toxicity after reirradiation, preclinical data consistently indicates substantial recovery in a time and dose-dependent manner for the central nervous system (both brain and spinal cord), corroborated by retrospective clinical studies [7], [8]. However, for other OARs, such as the bladder and gastrointestinal tract, there is no indication of recovery, while a time-dependent decrease in tolerance has been observed for the heart and kidneys [9].

The hypothesis that tumors acquire a specific radio-resistant phenotype, rendering reirradiation ineffective, has been derived from in vitro studies, although it currently still lacks substantial support from in vivo and clinical studies [10]. Instead, the response to and duration of initial radiotherapy, the treatment intent (whether palliative or curative), and the feasibility of administering a reasonable radiation dose respecting organs at risk (OAR) tolerances should guide decision-making. For symptom control, lower dose reirradiation with hypofractionated schedules is advisable, particularly for patients with a limited life expectancy.

To achieve longer-term local control and potentially cure, high-dose reirradiation with biologically equieffective doses comparable to the initial radiotherapy course is recommended to achieve reasonable local control. A distinction between local failure of the initially treated tumor and the occurrence of a new primary or a metastases is necessary for this decision making process, as it informs on the efficacy of the initial treatment. For local recurrences, higher doses than the initial course may be justified since the first radiation dose failed to ensure long-term local control. For new primaries or metastases the dose recommendation per standard of care might suffice. This is reflected in the definition of Typ I and Typ II reirradiation [1]. Of note, the time interval between first and second course of radiotherapy may have prognostic implications, i.e. the longer the interval the more favorable the outcome after reirradiation, as shown by Ward et al. in head and neck cancer patients and should be taken into account in the decision making process [11].

As per a recent consensus, recovery for OAR should be considered only for the CNS or spinal cord and not presumed for other OARs [1]. This approach does not preclude the safe delivery of high cumulative doses but suggests a more conservative approximation of the biological effects of accumulated doses, as understood through the linear-quadratic model, which is instrumental in calculating radiobiological equieffective doses for different dose and fractionation schemes [12].

### Key challenges and unmet needs

• **Patient Selection for High-Dose Reirradiation:** Identifying tumors who will respond to high-dose re-irradiation and quantifying the benefit for the individual patient.
• **Cumulative Radiation Dose Assessment:** Strategies for evaluating cumulative radiation doses, including estimating and accounting for uncertainty and tissue recovery
• **Predicting Side Effects and Tumor Radiosensitivity:** Utilizing genetic and non-invasive tests, such as advanced imaging, to identify patients at risk of side effects and to determine tumor radiosensitivity.

## Implementation of a clinical reirradiation workflow

Reirradiation is a complex treatment technique that requires a defined workflow to ensure a safe and practical approach, while addressing numerous uncertainties and limitations. The implementation of this workflow in the online adaptive MR-guided setting necessitates adjustments to accommodate the unique capabilities and challenges of this technique. Major challenges in re-irradiation include collecting and interpreting previous treatment information, treatment planning, defining critical OAR toxicities, and dose accumulation, each introducing a certain level of uncertainty that is challenging to quantify and account for.

Collecting previous treatment information involves technical, administrative, and data safety challenges. Limited resources for requesting and collecting previous treatment information and reliance on patients' recollections are common issues. Data availability may be constrained by the length of time since the patient's last treatment, regional documentation quality, and stringent data security laws hindering patient data transfer between countries, regions, and institutions. Full information of previous treatments and related imaging is recommended for use in dose accumulation, but not mandatory, and should not preclude reirradiation [1]. In cases where dose accumulation from previous treatment does not influence current treatment decisions, importing all previous information may be unnecessary [13]. This is particularly relevant in clearly palliative contexts or when the previous treatment is distant from the upcoming course.

Treatment planning techniques for reirradiation vary in complexity and implementation feasibility in commercial treatment planning systems. Many planners rely on trial and error to meet defined OAR tolerances, often necessitating multiple attempts to reach a clinically acceptable solution. Duffy et al. proposed a solution using a feasibility prediction tool and Pinnacle’s Auto planning function, where constraints were placed on structures generated in MIM from the accumulation of previous doses [14]. Their plans were found to be of higher quality than manually planned ones. McVicar et al. created a clinical tool for preparing dose distributions in BED for plan optimization, integrating a Matlab tool into the Varian Eclipse treatment planning system (TPS) [15]. They demonstrated success in meeting OAR tolerances for a lung cancer case. Murray et al. provided a solution in a research version of RayStation, adding previous dose distributions as a base dose for voxel-by-voxel optimization in EQD2 [16]. Their plans were mostly clinically acceptable and less likely to require relaxed OAR constraints. However, these solutions require additional technical capabilities not widely available or integrated into commercial TPS, which could limit the implementation of a reirradiation workflow. A survey found that clinics desire integrated tools within TPS for both treatment planning and dose accumulation [17].

A recent French national survey highlighted current practices in re-irradiation [18]. Many centers performing re-irradiations face similar challenges, with a quarter of respondents lacking the appropriate equipment for reirradiation, specifically software for dose accumulation. Paradis et al. described a Special Medical Physics consult as part of an implemented reirradiation workflow, where a physicist assesses case complexity and feasibility, aiding treatment planning efficiency [13]. Despite this feasible approach, no definitive guidelines exist for each step's definition or workflow implementation.

Hybrid MR-linacs, a recent innovation, facilitate MR-guided online adaptive radiation therapy (MRg-OART) and gated treatment delivery based on MR fluoroscopy. These features make MR-linacs a promising option for reirradiation treatment, though evidence on optimal implementation is limited. A notable difference in the MRg-OART setting is the need to prepare for online adaptations and daily assurance of meeting cumulative dose thresholds. Doty et al. presented a case report of pancreatic reirradiation using MRg-OART, achieving an escalated dose of 50 Gy in 5 fractions without acute or long-term grade 3 or greater toxicity, marking a significant advancement in reirradiation treatment in the MRg-OART setting [19].

### Key challenges and unmet needs

• **Evidence and Guideline Deficits:** Paucity of evidence or guidelines for reirradiation workflows, particularly in MRgRT.
• **Complexities in Treatment Planning:** The challenges in treatment planning and dose accumulation, compounded by indefinite intervals between reirradiation courses.

**Software Environment Limitations:** Difficulties posed by proprietary software environments in exchanging and integrating dose accumulation for planning.

## Technical aspects of dose accumulation

Robust evaluation of cumulative dose in the reirradiation setting requires mapping of previously delivered dose distributions onto the current anatomy as well as radiobiologically correct rescaling into equieffective doses prior to dose summation [1].

Mapping of dose distributions between imaging from multiple treatment courses is contingent on image registration providing locally accurate alignment of relevant anatomy − or at least alignment with sufficient accuracy to guide clinical decision making. This is particularly challenging in the reirradiation setting, where long time frames between radiotherapy courses, other cancer treatment modalities (such as surgery), and radiation-induced changes all may cause significant − and often dramatic − differences in anatomy [20]. Small retrospective in silico studies have demonstrated the value of deformable image registration (DIR) for reirradiation dose mapping (see e.g. [21], [22], [20], [23]), but there are still multiple limitations for clinically available algorithms, as recently reviewed by Murr et al [24]. In particular, these algorithms cannot realistically handle missing tissue and other non-continuous deformations (e.g. due to surgery or tumor shrinkage); often due to build-in algorithm regularization. Many algorithms in clinical use also depend on image intensity matching, which may not be constant in the reirradiation setting, e.g. due to radiation-induced tissue effects manifesting as radiographic changes. Despite these challenges, recent publications indicate that improvement in clinical algorithms is possible [25].

Once dose has been mapped to a reference anatomy − typically the imaging for the current treatment course − all dose distributions must be rescaled to equieffective dose (e.g. equivalent dose in 2 Gy fractions, EQD2) to account for fractionation effects prior to dose summation [12]. Note that it is a common misconception that this step is unnecessary for treatment courses with identical prescription dose per fraction. Optimally, equieffective dose rescaling is performed on a voxel-by-voxel level for the full 3D dose distributions, followed by 3D dose summation, especially if volumetric dose measures are to be evaluated [26], [27]. However, this functionality is not always available in clinical treatment planning systems (TPS), representing a major barrier to safe evaluation of cumulative reirradiation dose distributions [17].

The practical implementation of the steps outlined above − image registration for dose mapping and equieffective dose rescaling followed by dose summation − results in a complex workflow, with many opportunities for intercentre variation. A recently published multicentre study demonstrated significant discrepancy in estimated cumulative dose for reirradiation cases evaluated by 24 observers via their individual clinical pathways [28]. There is a considerable and urgent need for international guidelines for reirradiation dose accumulation methodology.

Each of the dose accumulation steps are associated with a number of uncertainties, even when conducted in a standardized manner, ranging from image registration imprecision and inherent limitations to uncertainty in radiobiological parameter values (including tissue recovery factors) for equieffective dose rescaling. This impacts the clinical confidence in dose accumulation and consequently in the utilization of high-dose reirradiation. Although a few papers have proposed technical solutions to incorporate uncertainties into dose summation and treatment planning, there are no commercially available tools available nor any clear scientific consensus on how uncertainties should be assessed and evaluated [29], [30], [31]. This represents another major and ongoing challenge.

### Key challenges and unmet needs

• **Image Registration Limitations:** Addressing the deficiencies in clinically available image registration algorithms in the reirradiation settings, including evaluation of registration quality and uncertainties.
• **Availability of Tools for Dose Accumulation:** The scarcity of user-friendly tools for radiobiologically appropriate 3D dose accumulation in most commercial treatment planning systems.
• **Lack of Standardized Dose Accumulation Protocols:** The absence of international standards for technical dose accumulation methodology and reporting of associated uncertainties.

## Reirradiation with protons

Protons and other charged particles primarily deposit their energy at a specific depth, thereby minimizing low to moderate doses to adjacent OARs. The rapid dose falloff beyond the target is pivotal in reducing OAR toxicity in radiation therapy [32]. The criteria for selecting proton therapy in primary radiotherapy vary internationally and are largely consensus-driven. Often, they are based on local guidelines, comparisons of photon and proton treatment plans, the use of a normal tissue complication model-based approach, or cost-effectiveness modeling. However, high-quality evidence is still awaited for many tumor types [33]. Indications for proton therapy in re-irradiation also vary internationally[34], [35]. The literature on proton reirradiation is mainly characterized by retrospective studies, single-institution reports, small sample sizes, and the absence of direct comparisons with equivalent photon therapy. In silico analyses in both the primary and reirradiation settings suggest dosimetric benefits [36], [37], [38]. Despite the lack of high-quality studies, proton therapy is often considered an attractive option for reirradiation when reduction in integral dose is warranted.

Proton reirradiation has been reported for various tumor types, predominantly in head and neck, central nervous system, or thoracic tumors. A systematic search across all tumor sites yielded 3 prospective studies, 23 retrospective studies, 7 technical or in silico analyses, and 19 reviews/expert opinions [39]. A 2022 review on head and neck cancer, which included only retrospective studies on proton therapy, concluded that “proton reirradiation appears promising and may allow for higher doses delivered with less toxicity” [40]. In the context of gliomas and glioblastomas, a 2022 review included reirradiation. It identified only one study with prospectively collected data, showing higher overall survival and increased toxicity with proton therapy compared to photon studies, potentially due to higher radiation doses in the proton study [41]. Another prospective trial on glioblastoma, not included in the review, found preserved health-related quality of life after proton reirradiation, with no comparison to photon therapy [42]. A review and *meta*-analysis on reirradiation for lung tumors included 3 retrospective and one prospective proton study. Overall, proton reirradiation caused similar toxicity rates compared to conventional radiotherapy, often administered at lower or palliative doses, and thus not directly comparable. A subsequent retrospective study comparing outcomes and toxicity of proton and photon therapy found similar results for the two modalities [43]. Preclinical studies have suggested radiobiological benefits of proton therapy related to differences in relative biological effectiveness/linear energy transfer (RBE/LET), hypoxia sensitivity, and immunogenicity factors, which could also be advantageous in reirradiation but require further research [44], [45], [46].

Given the beneficial physical dose distribution, there is a clear rationale for considering proton therapy in reirradiation planning. Patients should be categorized based on obvious benefit, potential benefit, and no benefit. If evaluation of imaging and clinical findings indicates that proton therapy results in improved target coverage, relevant dose escalation, or decreased dose to a critical target structure, the benefit is clear. If the assessment suggests a potential benefit, comparative treatment plans with protons and photons may assist in decision-making. However, determining potential benefit is often challenging, as data on cumulative constraints and OAR recovery are sparse. Additionally, these comparisons are often complicated by difficulties in image registration and anatomical changes between primary and re-irradiation scans.

### Key challenges and unmet needs

• **Patient Selection:** Distinguishing patients who will clearly benefit from proton reirradiation.
• **Dose-Response Relationships in Reirradiation:** Improve knowledge on dose–response relationships for normal tissue damage, especially concerning low-dose area to guide selection of patients for proton therapy.
• **Proton Therapy Knowledge Gaps:** Limited understanding of the radiobiological and immunogenetic advantages of proton therapy.

## Reirradiation with brachytherapy with focus on gynecological cancer

Good brachytherapy techniques ensure positional stability of the target relative to the applicator and allow daily adaptation to the CTV without the need for ITV and PTV margins for internal motion and setup uncertainties [47]. Combined with the steep dose fall-off from the source, these characteristics make brachytherapy an ideal modality for reirradiation in selected gynecological tumors. A recent literature review by the American Brachytherapy Society (ABS) demonstrated favorable results [48]. Most reports focused on interstitial HDR brachytherapy for recurrent cervical cancer, followed by endometrial and mixed tumor types. A wide variation in doses and fractionation regimens was used. After a median follow-up of 1 to 5 years, local control rates were approximately 70 to 90 % at 1 year, 45 to 70 % at 3 years, and 45 to 70 % at 5 years. Corresponding overall survival rates were around 80 %, 50 to 70 %, and 40 %. Late grade 3 to 4 toxicity ranged from 0 % to 43 %, with most authors reporting over 15 % of serious side effects. Some data suggested improved outcomes with reirradiation doses exceeding 40 to 50 Gy. Long intervals between the initial radiation and reirradiation, and the size of recurrent tumors less than 2–4 cm, appeared to predict better outcomes [48]. Since the ABS review, at least 7 additional retrospective studies have been published, including two large reports on image-guided adaptive brachytherapy (IGABT) [49], [50].

To analyze the reporting patterns, we searched the literature on gynecological reirradiation with brachytherapy from 1990 to 2023 and identified 36 series, collectively reporting on 1267 patients (Supplementary Table 1). Of these, 889 (70 %) received brachytherapy for reirradiation (Table 1). There was considerable variation in sample sizes, tumor entities, techniques, doses, and fractionations among published studies, with most not reporting cumulative nominal or EQD2 doses of primary treatment and reirradiation.

Intracavitary (IC) brachytherapy alone is suitable only for rare patients with central recurrences and a depth of invasion less than 0.5–0.7 cm from the vaginal wall. Deeper lesions should be re-treated with transperineal and/or transvaginal interstitial (IS) or combined IC/IS approaches. Peripheral pelvic recurrences are typically more suitable for re-treatment with external beam radiotherapy (EBRT), particularly stereotactic body radiotherapy (SBRT). Our review of 36 studies showed that 19 (53 %) used HDR only, 11 (31 %) used (V)LDR only, and 5 (14 %) used both approaches. There is no known clinical advantage of a specific dose rate, but practical differences may exist. HDR and PDR techniques allow post-implant compensation for suboptimal implant geometry by adjusting dwell times and positions. Channel loading optimization near OARs enables personalized fine-tuning of the dose in critical regions. Temporary implant geometry inadequacies can be corrected in subsequent applications. Gynecological radiation oncologists commonly have extensive experience with HDR brachytherapy in the primary setting. HDR poses less radiation protection challenge, carries no risk of source migration, and may be more biologically effective in adenocarcinomas with lower alpha/beta ratios. The major advantage of LDR over HDR is delivering a curative dose with a single implant. Based on the linear-quadratic model, it could also offer a wider therapeutic window between tumor and OAR effects in most patients. Regardless of the dose rate, clear recommendations on brachytherapy dose and fractionation are not possible due to limited evidence from small, heterogeneous retrospective series and expert opinions. While some authors found improved outcomes with reirradiation doses of over 40 to 50 Gy to the target volume, establishing dose–effect relationships was not successful [48]. Treatment intent, life expectancy, comorbidities, past cumulative EQD2 for the tumor and OAR, side effects of past treatment, and duration of tumor response need individual consideration when deciding planning aims and dose constraints. Pre-brachytherapy cystoscopy and proctosigmoidoscopy may be beneficial and can guide decisions. Existing OAR dose constraints for the primary setting should be respected whenever possible, but are often intentionally exceeded, especially when reirradiation follows a full course of EBRT plus brachytherapy. In such cases, over 15 % of grade 3 late side effects can be expected, underscoring the need for high-risk informed consent and patient participation in shared decision-making. Doses and fractionations reported in the literature exceed the scope of this summary and are detailed in the review by Sturdza et al. [48].

The scarcity of publications, small sample sizes, heterogeneity of patient cohorts and treatments, and inconsistent/incomplete reporting of brachytherapy for reirradiation are major challenges. For more reliable future insights, standardized prospective reporting based on the ESTRO EORTC consensus [1] should be adopted in clinical routine and real-life observational studies, ideally building on the experience and network of the EMBRACE research group.

### Key challenges and unmet needs

• **Dose Summation Methods:** Evaluating methods like simple summation versus registration techniques, mindful of dose over/underestimation risks.
• **Uncertainty Assessment:** Assessing uncertainties in each procedural step, from indication to brachytherapy technique, contouring, image registration, dose optimization, and summation.
• **Consensus Definition and Reporting Guidelines:** Promoting the dissemination and application of the ESTRO EORTC consensus on reirradiation for brachytherapy.

## Reirradiation in combination with hyperthermia

Moderate hyperthermia, heating a tumor to 41 to 43 °C for one hour, is a very potent radiosensitizer and induces little toxicity, as multiple clinical studies have shown. There is a strong rationale to combine hyperthermia with radiotherapy, especially for reirradiation, because the radiation dose is usually limited by the previous radiotherapy. Additionally, patients often have limited further systemic options or cannot tolerate additional systemic therapy. In this case, hyperthermia may compensate for the missing radiosensitizing effect of chemotherapy. Moderate hyperthermia delivered with microwaves and adequate thermal dose can be applied to tumors throughout the body, except for tumors in the brain and lungs, due to physical limitations in these anatomical sites.

In a prospective study, 122 patients with superficial tumors, heated to a thermal dose of more than 10 cumulative equivalent minutes at 43 °C (CEM 43 °C) T90 measured by invasive thermometry, were randomized between hyperthermia and radiotherapy versus radiotherapy alone [51]. The complete response rate was 66.1 % in the hyperthermia arm and 42.3 % in the no-hyperthermia arm. The odds ratio for complete response was 2.7 (95 % CI, 1.2 to 5.8, p = 0.02). Previously irradiated patients (mostly breast/chest wall recurrences) had the greatest incremental gain in complete response: 23.5 % in the no-hyperthermia arm versus 68.2 % with hyperthermia. Additionally, a Dutch study in patients with locoregional breast cancer recurrences treated with postoperative radiotherapy in combination with hyperthermia showed the impact of temperature [52]. This retrospective study analyzed 112 patients who received reirradiation with 46 Gy/2 Gy or 32 Gy/4 Gy combined with 4–5 weekly hyperthermia sessions with invasive thermometry. Patients were divided into low versus high thermal dose groups based on the best session with the highest median CEM 43 °C T50 < 7.2 min and ≥ 7.2 min, respectively. Three-year locoregional control was 74 % in the low thermal dose group and 92 % in the high thermal dose group (p = 0.008). Palliative reirradiation with only 4 Gy once weekly up to a total dose of 20 Gy in combination with water-filtered infrared hyperthermia showed great promise in patients with recurrent breast cancer [53]. In this treatment regimen, 201 patients with recurrences of varying local extent achieved a complete or partial response in 95 % of cases during follow-up. This schedule's advantage is its repeatability. Reirradiation combined with deep hyperthermia also showed promise in locally recurrent rectal cancer in the prospective phase II HyRec study [54]. Of the 105 patients in this study, 16 had locally recurrent rather than primary rectal cancer. These patients received 45 Gy/1.8 Gy + 5FU/Oxaliplatin + twice-weekly deep hyperthermia, and a complete pathological response was observed in 3 of 8 (38 %) recurrent tumors that could be resected after trimodal treatment, which is extremely promising despite the small number of patients.

### Key challenges and unmet needs

• **Hyperthermia as an Immune Modulator:** Exploring the effects of combining with immune checkpoint inhibitors or other agents.
• **Standardization Needs:** Addressing the lack of standardization in hyperthermia delivery, with harmonized treatment and quality assurance protocols.
• **Access to Combined Treatments:** The challenges in ensuring timely intervals between reirradiation and hyperthermia, which are critical in centers offering both treatments.

## Head and neck cancer reirradiation

Historically, the Vokes regimen, which includes 60 Gy administered as 2 Gy per fraction, 5 fractions per week, over 11 weeks (with a week on and a week off), was combined with 5FU (800 mg/m^2^) and hydroxyurea (1.5 g/day) [55]. In previously irradiated patients with recurrent head and neck cancer for palliation, a randomized phase III GORTEC trial 98–03 compared methotrexate alone (40 mg/m^2^/week) to reirradiation combined with methotrexate, but found no difference in overall survival [56].

For curative intent in previously irradiated patients with recurrent head and neck cancer, either definitive chemoradiotherapy or postoperative chemoradiotherapy can be implemented. Generally, definitive chemoradiotherapy is associated with increased toxicity, with 2-year overall survival rates ranging from 15 % to 25 % [57], [58], [59]. In the postoperative chemotherapy and reirradiation setting, a randomized phase III trial compared surgery alone to 60-Gy postoperative CRT in 130 patients (65 patients in each arm) [5]. The trial demonstrated significantly better locoregional control in the postoperative chemoradiotherapy arm compared to surgery alone (p < 0.0001), but overall survival was similar (p = 0.50). The incidence of grade 3–4 toxicity was substantially higher in the postoperative chemoradiotherapy arm (39 %) versus the surgery alone arm (10.5 %).

The introduction of intensity-modulated radiotherapy (IMRT) has improved toxicity outcomes. Generally, an elective radiotherapy volume receiving 50 Gy for head and neck cancer is approximately 1.5 L; however, this volume can be reduced to 800–900 cc with IMRT, potentially resulting in improved outcomes [60]. Hyperfractionation has also been investigated. In a phase II randomized study including postoperative CRT after salvage surgery in head and neck cancer patients using 3D-conformal radiotherapy or IMRT, 60 Gy in 5 weeks (1.2 Gy/fraction, 2 fractions/day) and cetuximab was compared to the Vokes regimen (60 Gy in 11 weeks plus chemotherapy) [61]. However, no significant difference was found in terms of outcome (neither overall survival nor progression-free survival). The grade 3–4 toxicity was also comparable.

A multicenter Chinese phase III trial in advanced recurrent nasopharyngeal cancer patients, using IMRT reirradiation in both arms (chemotherapy was not obligatory), found that patients in the hyperfractionation group had a better overall survival (p = 0.014) than those receiving conventional fractionation (in 144 patients, 72 patients per arm, 64.8 Gy in 1.2 Gy/fraction vs. 60 Gy in 2 Gy/fraction) [62]. Grade 3 late toxicity was lower in the hyperfractionation group (34 %) compared to the conventional fractionation group (57 %).

A single arm phase II study reported promising results with stereotactic reirradiation and cetuximab in 60 patients with inoperable recurrent, or new primary head and neck cancer in a previously irradiated area [63]. The median overall survival was 11.8 months (47.5 % at 1-year), and the rate of grade 3–4 toxicity was acceptable. Another single arm phase II study, focussing on SBRT and cetuximab, showed a median overall survival of 10 months (40 % at 1-year), again with acceptable toxicity [64]. Based on these two prospective studies, good indications for SBRT were recurrence time greater than 6 months following initial radiotherapy, a second primary tumor, and inoperable lesions less than 6 cm in diameter.

In centers where the equipment is available, particle therapy reirradiation, including protons or carbon ions, represents a promising option [65], [66]. In the postoperative reirradiation setting, brachytherapy may be a promising treatment option.

An interesting approach has been followed by Martinez-Fernández and colleagues where they employed perioperative brachytherapy in select patients suitable for surgical resection and applied a different cumulative dose in case of an R0 or R1 resection. Despite careful patient selection and the targeted radiation approach, grade 3 and higher toxicity was considerable (approx. 50 %) with a five year local control rate of 55 %. According to the CHUV experience, using a median of 3 catheters inserted during surgery and using micro-selectron high-dose-rate brachytherapy and a median dose of 48 Gy in median 3 Gy/fraction, twice-a-day, in 44 patients with a median follow-up period of 23 months, 50 % 3-year local control with acceptable toxicity was obtained (personal communication, unpublished data).

Another emerging treatment option is the combination of deep hyperthermia and reirradiation in recurrent head and neck as either definitive or postoperative treatment [67].

In summary, if a patient is suitable for surgery (approximately 20 % of patients), the preferred treatment for locoregional recurrence in head and neck cancer is surgical resection and should be offered to and discussed with the patient. Otherwise, reirradiation with a curative-intent radiation dose (≥ 60 Gy) is a feasible option and should be assessed. To guide in patient selection and the selection of the appropriate radiotherapy technique the 5 question based executive summary of the American Radium Society may serve as a guide until better evidence is available [11].

### Key challenges and unmet needs

• **Patient selection for optimal radiation technique:** Identifying tumor and patient characteristics that help select the optimal treatment techniques from VMAT EBRT, particles or brachytherapy.
• **Target volume determination:** Investigating target volume definition to determine the need for elective volume irradiation and the possibility to reduce treatment margins.
• **Combination therapy:** Enhancing local control by combination with systemic therapies and/ or hyperthermia to reduce radiation dose and thus the potential for reirradiation induced side effects.

## Thoracic reirradiation

In the pre-immunotherapy era, to our knowledge, there was only one prospective study on high-dose reirradiation available [68]. This study included 59 evaluable patients with recurrent tumors in the lung parenchyma following previous high-dose radiotherapy. The recurrences were diagnosed a median of 30 months after the initial treatment. All patients underwent high-dose SBRT. Dyspnea of grade 2 and 3 occurred in 8 % of patients before treatment and increased to 14 % after treatment. Additionally, 14 % of patients experienced grade 2 chest wall pain, and 5 % developed grade 2 plexopathy. The estimated 5-year rates for local, regional, and distant failure were 5.2 %, 10.3 %, and 22.4 %, respectively. The estimated progression-free survival was 46.2 % at 3 years and 41.1 % at 5 years. The overall survival rates were 63.5 % at 3 years and 56.5 % at 5 years. This study supports high-dose reirradiation with SBRT in selected patients with a local recurrence or a second primary cancer, particularly when there is a long interval between the primary treatment and when the overlap between high-dose areas is confined to the lung parenchyma.

Fifty-two patients completed reirradiation without immunotherapy using protons for non-small cell lung cancer (NSCLC), with 67 % receiving concurrent chemotherapy [69]. The median radiation dose was 66 GyE. After a median follow-up of about 8 months, the one-year progression-free survival was 58 %. Twelve percent of patients developed severe late toxicity, and six patients died due to treatment-related side effects. In a retrospective series of 22 patients reirradiated with intensity-modulated proton therapy (mostly 60 GyE/30 fractions), 10 received durvalumab as well [70]. The one-year progression-free survival rate was 45 %, with 9 % experiencing grade 3 late toxicity.

Only small series of salvage surgery after high-dose radiotherapy have been reported in the pre-immunotherapy era [71]. These series are diverse, and the selection criteria often unclear, but it appears that in highly selected patients, resection, mainly lobectomy, may be associated with a 5-year overall survival rate of 10–50 %, with about 40 % morbidity and 4 % surgical mortality. Only a few case reports on salvage surgery after chemoradiotherapy and durvalumab are available, showing that this approach may be feasible [72], [73]. In a retrospective series of 50 patients treated with pembrolizumab, with or without chemotherapy, for recurrent disease after concurrent chemoradiotherapy and durvalumab, the one-year progression-free survival rate was 25 % [74].

All patients eligible for high-dose reirradiation should undergo thorough staging, and every effort should be made to obtain pathology of the new tumor. Most patients develop distant metastases. SBRT is recommended for reirradiation when there is only an overlap of high-dose regions in the lung parenchyma. Overlap with mediastinal structures should be avoided as much as possible, and no repair of previously irradiated organs should be assumed. In all other cases, reirradiation should only be considered when there are no alternatives such as surgery​​.

### Key challenges and unmet needs

• **Dose De-Escalation Strategies:** Investigating strategies to maintain control while reducing toxicity, such as radiosensitization through combined immunotherapy.
• **Improving Distant Disease Control:** Enhancing control with better systemic agents.
• **Determining Intrinsic Radiosensitivity:** Using genetics and other non-invasive tests, including novel imaging modalities to determine which patients are prone to developing radiation-induced side effects.

## Prostate cancer reirradiation

Prostate cancer is the third most common cause of cancer mortality in men, following lung and colorectal cancers [75]. The recurrence rate after primary EBRT varies depending on the D'Amico risk group. The prostate is the most common site of recurrence across all risk groups, even in patients treated with higher doses. In a comprehensive patient series treated with doses equal to or greater than 76 Gy, intraprostatic relapse ranged from 4 % to 15 %, according to the D'Amico group. Half of the patients with clinical relapse presented with isolated prostate disease [76].

Local salvage treatment is offered to a very small percentage of patients, and androgen deprivation therapy (ADT) is the most common treatment. For instance, in the study by Tran et al., ADT and observation were employed in 46 % and 49 % of the patients, respectively, but ADT was eventually used in half of the patients initially under observation [77].

Most intraprostatic relapses occur at the same location as the dominant index lesion before the initial EBRT. In extensive series of salvage surgery, over 60 % of patients present with unifocal recurrence, suggesting that focal treatment could have been proposed in more than half of the cases [78]. Multiparametric MRI is pivotal in local evaluation of these patients, and the Prostate Magnetic Resonance Imaging for Local Recurrence Reporting (PI-RR) should be utilized [79].

A recent *meta*-analysis of local salvage therapies by Valle et al. highlighted the paucity of published prospective series, regardless of the treatment modality (surgery, cryotherapy, high intensity focused ultrasound, low and high dose rate brachytherapy, SBRT) [80]. Recurrence-free survival rates, regardless of salvage treatment, are around 50 % at 5 years. Brachytherapy and SBRT were less toxic to the genito-urinary tract than surgery, and the rate of severe gastro-intestinal toxicity was low. In a *meta*-analysis of salvage SBRT, more than half of the patients received focal treatment; severe toxicity was low, but with a short follow-up [81]. Higher doses were associated with more severe toxicity and longer relapse-free survival. Focal SBRT was not linked to lower relapse-free survival. The most commonly used regimen was 6 fractions of 6 Gy, as selected in the only ongoing phase 2 study of salvage SBRT in Europe [82]. Delphi consensus on salvage brachytherapy and SBRT have been published, yet consensus was not reached on all of the questions: e.g.there is no consensus on the optimal volume (focal or whole gland) for brachytherapy, while experts have selected focal SBRT based on MRI plus adaptive margin [83], [84].

Patients should be thoroughly informed about the advantages and potential disadvantages of salvage radiotherapy. The selection of these patients must be rigorous, based on factors such as the risk group before the first radiotherapy, PSA doubling time at recurrence, life expectancy and health status, urinary function based on the International Prostate Symptom Score (IPSS) score, interval since previous radiotherapy, and absence of late severe toxicity.

Intraprostatic recurrence must be confirmed with a biopsy; random whole-gland and targeted biopsies are preferred. Patients should be evaluated with PSMA-PET and multiparametric MRI. Due to the low level of evidence, inclusion in prospective trials or registries is highly recommended.

### Key challenges and unmet needs

• **Patient Selection:** Determining the most beneficial patients for salvage reirradiation and incorporating biological and radiological biomarkers.
• **Target Volume Optimization:** Defining the optimal target volume (whole gland versus focal treatment, role of multiparametric MRI, optimal CTV margins).
• **Organ-at-Risk Dose Constraints:** Establishing optimal dose constraints for organs at risk.

## Bone metastases reirradiation

Between 11 to 42 % of patients with bone metastases require reirradiation, with approximately 60 % experiencing pain relief after palliative radiotherapy [85]. The majority of patients (75 %) are retreated due to recurrent pain, while others receive retreatment due to insufficient (10 %) or no pain response (15 %) [2], [85]. Despite single and multiple fractionation schedules being equally effective for pain relief, reirradiation rates are significantly higher after single fraction radiotherapy compared to multiple fraction schedules: 20 % versus 8 %, respectively [86]. The Dutch Bone Metastases Study, the largest randomized trial comparing single with multiple fraction radiotherapy for bone metastases patients, conducted an in-depth analysis of the reasons for reirradiation [87]. They found the average duration of pain response was 18 weeks after single fraction radiotherapy and 19 weeks after multiple fractions. Pain recurrence occurred in 51 % of patients after single fraction treatment and 47 % after multiple fractions. However, single fraction patients were more frequently, earlier, and at lower pain scores retreated, indicating a tendency among physicians to retreat patients more readily after single fraction radiotherapy.

The most suitable reirradiation regimen was explored in the largest randomized trial in this field [2]. In this study, patients (n = 850, more than 4 weeks from initial radiotherapy, median 4 months) were randomized between a single 8 Gy fraction or 20 Gy in multiple fractions (5 or 8 fractions, depending on previous dose and target location). The primary endpoint was the overall response rate at 2 months. The study faced challenges in assessment, with only 62 % of patients evaluable at 2 months. In the intention-to-treat population, 118 of 425 patients (28 %) receiving a single fraction and 135 of 425 patients (32 %) receiving multiple fractions experienced pain relief. However, in the per-protocol population, the upper boundary of the 95 % confidence interval for this difference exceeded the non-inferiority margin. Early side effects like vomiting and diarrhea were more frequent after multiple fractions. A secondary analysis of this trial indicated that patients responding to reirradiation experienced better quality of life scores and less functional interference from pain [88]. Notably, pain response to previous radiation and previous radiation fractionation schedule were not associated with pain response, demonstrating the benefits of reirradiation even for patients who did not respond to initial therapy.

As the number of advanced cancer patients with bone metastases increases, and systemic therapies improve, a significant proportion of patients will live for years after metastasis diagnosis. Some of these patients may benefit from reirradiation with SBRT, initially developed for reirradiation purposes [89]. A systematic review of 9 prospective and retrospective cohort studies reported a 1-year local control rate of 66 to 90 % and a pain control rate of 65 to 81 %, although assessment tools varied [90]. The largest multi-institutional pooled analysis for spinal metastases (n = 215) showed a 13 % local failure rate and an 83 % 1-year local control rate, with the majority of patients (74 %) reporting improved pain and low toxicity rates [91]. No comparative or randomized trials have been published comparing reirradiation using conventional radiotherapy or SBRT.

In daily clinical practice, decision-making remains complex because many patients with bone metastases seem adequately treated with single fraction conventional radiotherapy. Trade-offs might exist between efficacy and inconvenience or toxicity. For patients with a limited number of metastases or radioresistant tumors, reirradiation using SBRT may be the preferred treatment option. The HyTEC recommendations for reirradiation SBRT delivered in 1 to 5 fractions provide guidance for safe treatment [92].

### Key challenges and unmet needs

• **Fractionation Schemes:** The rise of ultra-short schemes as standard, though certain subgroups might benefit from longer regimens.
• **SBRT versus Conventional Therapy:** Confirming the possible superiority of SBRT over conventional therapy through trials or registries.
• **Safe Delivery of Multiple Courses of Radiotherapy:** Ensuring safe delivery of second or third reirradiation courses in the context of more effective systemic treatments.

## Abdominal reirradiation

Reirradiation in the abdominal area requires careful evaluation of various factors, including tumor type, previous treatment history, treatment interval, and specific patient considerations. For certain cancers like colorectal tumors, reirradiation doses around 40 Gy have been shown to provide reasonable local control [93], [94]. Higher doses may be needed, especially when radical surgery is not an option [95]. Conversely, pancreatic cancer often necessitates higher doses for effective local control, but the potential toxicity risks must be carefully weighed [96], [97]. The challenge in determining safe doses is confounded by the limited data on long-term normal tissue tolerance [98]. Complications after reirradiation predominantly involve bowel toxicity, ranging from bleeding to obstruction with risk of fatal outcomes. These severe complications can emerge rapidly after treatment, sometimes within a month [99], [100]. Risk factors include radiation doses from previous radiotherapy courses, short intervals between treatments, pre-existing bowel damage, and cumulative intestinal radiation doses [97], [101]. Such findings align with animal studies that link radiation dose with mucosal damage [102].

Abdominal reirradiation decisions should be tailored per individual patient. Safe cumulative doses for abdominal reirradiation vary based on factors such as tumor type, treatment goals, OAR involvement, previous treatments, treatment interval, and patient comorbidities.

Detailed treatment planning can assist in mitigating risks, but considerable uncertainties with regards to cumulative doses need to be considered when deciding on the final prescription dose and the potential toxicity risk for intestinal OARs. Minimizing severe toxicity can be achieved by adhering to dose constraints for organs like the stomach and bowel. A minimum interval of 4 months since the last radiation treatment is recommended, along with no history of gastrointestinal ulcers, as confirmed by endoscopy. Employing advanced methods, like SBRT and MR-guided radiotherapy (MRgRT), may enhance the safety of reirradiation.

### Key challenges and unmet needs

• **Clinical Evidence and Biomarker Shortfalls:** The need for robust clinical evidence, biomarkers for patient selection, and data on long-term normal tissue tolerance in abdominal re-irradiation.
• **Challenges in Dose Accumulation:** Addressing reproducible dose accumulation due to intra- and interfraction bowel motion affecting accuracy and precision dose prediction to target volumes and OARs.
• **Complex Decision-Making:** Navigating complex decisions balancing risks, benefits, and goals (curative versus palliative), especially when radical surgical options are available, necessitating multidisciplinary collaboration.

## CRediT authorship contribution statement

**Nicolaus Andratschke:** Conceptualization, Writing – original draft, Writing – review & editing. **Jonas Willmann:** Writing – original draft, Writing – review & editing. **Ane L Appelt:** Writing – original draft, Writing – review & editing. **Madalyne Day:** Writing – original draft, Writing – review & editing. **Camilla Kronborg:** Writing – original draft, Writing – review & editing. **Mariangela Massaccesi:** Writing – original draft, Writing – review & editing. **Mahmut Ozsahin:** Writing – original draft, Writing – review & editing. **David Pasquier:** Writing – original draft, Writing – review & editing. **Primoz Petric:** Writing – original draft, Writing – review & editing. **Oliver Riesterer:** Writing – original draft, Writing – review & editing. **Dirk De Ruysscher:** Writing – original draft, Writing – review & editing. **Joanne M Van der Velden:** Writing – original draft, Writing – review & editing. **Matthias Guckenberger:** Writing – original draft, Writing – review & editing.

## Declaration of competing interest

The authors declare that they have no known competing financial interests or personal relationships that could have appeared to influence the work reported in this paper.
