# Supplementary material for: Reirradiation − still navigating uncharted waters?
Source: Clin Transl Radiat Oncol. 2024 Oct 2;49:100871. doi: 10.1016/j.ctro.2024.100871 (PMC11497423; doi:10.1016/j.ctro.2024.100871)
Supplement: Supplementary Data 1 [file mmc1.docx]

# Supplementary material

**Table 1:** Patterns of reporting in 36 retrospective studies on gynecological reirradiation with brachytherapy, published between 1991 and 2023. ^a^Implicit reporting: frequency derived from the text using the methodology according to Andratschke et al.[[1]](https://paperpile.com/c/sWkQqW/RRNT) Full literature list is available by request to the authors.

| **Parameter** | **N of studies reporting** | **%** |
| --- | --- | --- |
| **Tumor site** | 36 | 100 |
| **Histology** | 29 | 81 |
| **Initial treatment** |  |  |
| Surgery | 31 | 86 |
| Chemotherapy | 19 | 53 |
| Radiotherapy method | 35 | 97 |
| Radiotherapy dose | 26 | 72 |
| Radiotherapy fractionation | 13 | 36 |
| Side effects of Radiotherapy | 1 | 3 |
| **Interval to re-irradiation** | 23 | 64 |
| **Brachytherapy for recurrence** |  |  |
| Reirradiation type^a^ | 36 (100) | 100 |
| Intent | 6 (17) | 17 |
| Technique | 36 (100) | 100 |
| Dose rate | 36 (100) | 100 |
| Fractionation | 34 (94) | 94 |
| BT planning |  |  |
| Imaging for planning | 36 (100) | 100 |
| 2D vs. 3D planning | 34 (94) | 94 |
| Target V aims | 10 (28) | 28 |
| OAR constraints | 8 (22) | 22 |
| Target V dose | 34 (94) | 94 |
| OAR dose | 26 (72) | 72 |
| **Treatment sum**** |  |  |
| Cumulative dose | 14 (39) | 39 |
| Registration | 13 (36) | 36 |
| EQD2 | 24 (67) | 67 |
| **Outcome** |  |  |
| Median follow-up | 31 (86) | 86 |
| Follow-up method | 12 (33) | 33 |
| Local control | 32 (89) | 89 |
| Overall survival | 27 (75) | 75 |
| Late side effects | 33 (94) | 94 |
| Quality of life | 0 (0) | 0 |
